# Anaphylactic Shock During Pulmonary Hydatid Cyst Surgery

**DOI:** 10.5812/aapm.16725

**Published:** 2014-06-23

**Authors:** Shaqayeq Marashi, Vahideh Sadat Hosseini, Alireza Saliminia, Amirabbas Yaghooti

**Affiliations:** 1 Departmant of Anesthesiology, Tehran University of Medical Sciences, Tehran, IR Iran

**Keywords:** Hydatid Cyst, Anaphylactic Shock, Surgery, Lung

## Abstract

**Introduction:**

Hydatid cyst is a parasitic disease caused by a tapeworm Echinococcusgranulosus. Humans are accidental hosts and infected after digestion of foods contaminated to fecal matter of definite hosts. The most affected organs are liver and lungs. Rupture of cyst (spontaneous rupture or rupture due to trauma or surgery) can cause anaphylactic reactions. Even considered as a rare event during anesthesia, it can be life threatening with the manifestations of severe hypotension and circulatory shock. Thus, immediate and proper treatment is necessary .

**Case Presentation:**

We report a case of anaphylactic shock during surgery of pulmonary Hydatid cyst in a 42 year old woman and its management.

**Conclusions:**

During the surgery of hydatid cyst, any hemodynamic instability should raise the suspension of anaphylaxis and early resuscitation should be instituted.

## 1. Introduction

Hydatid cyst is a parasitic disease caused by a tapeworm *Echinococcus granulosus* (1-5). The disease has a worldwide distribution, but there are some endemic regions such as Australia , America , New Zealand, Africa and Asia .The Liver is the most common affected organ and the lungs stand in second position ( both together account for 90% of the cysts) (4). Other possible affected organs are central nervous system, skeletal muscles, spleen and kidneys (4). Humans are intermediate hosts and are accidentally infected after digestion of contaminated food (6). Growing rate of hydatid cyst is slow (4) and it is usually asymptomatic in early stages, but gradually, it can cause obstruction or its rupture may create allergic reactions (1). The spectrum of clinical symptoms depend on various factors such as Specific organ which is involved; Size of cyst; Effect of cyst on adjacent organ structures; and Complications of cyst such as rupture and so on (4).

Pulmonary hydatid cyst can be asymptomatic; however, presenting signs and symptoms may include: chest pain, chronic cough, expectoration, dyspnea, hemoptysis, pneumothorax, parasitic lung embolism and eosinophilic pneumonitis. Rupture of cyst into bronchial tree can cause asthma like symptoms, symptoms of anaphylaxis and fever (4). The cyst has a variable size ranges between 1 cm and 15 cm (4). Incidence of complications is about 1/5000 to 1/20,000 procedures and mortality rate is about 3%-6% (3). Anaphylactic reactions due to ruptured pulmonary hydatid cyst are rare (3) and anaphylaxis during anesthesia is not common, but if anaphylaxis happens, the presentation varies from urticaria and conjunctival edema to life threatening circulatory shock (1-3, 6, 7). The reactions usually occur in the first 1 to 15 minutes after leakage of cyst fluid (6).We report a case of anaphylactic shock during pulmonary hydatid cyst surgery.

## 2. Case Presentation

**Figure 1.**
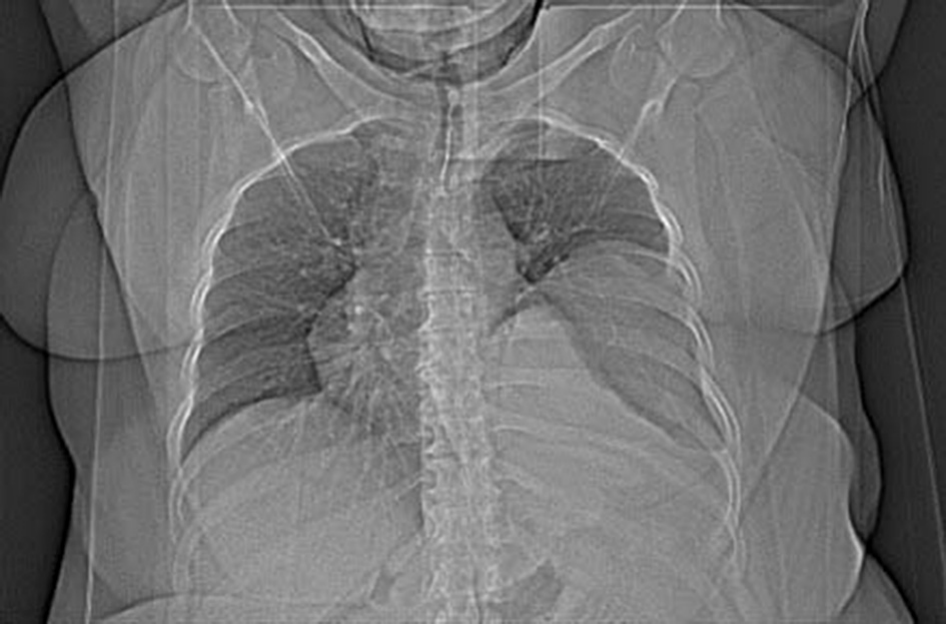
Chest CT-Scan

**Figure 2.**
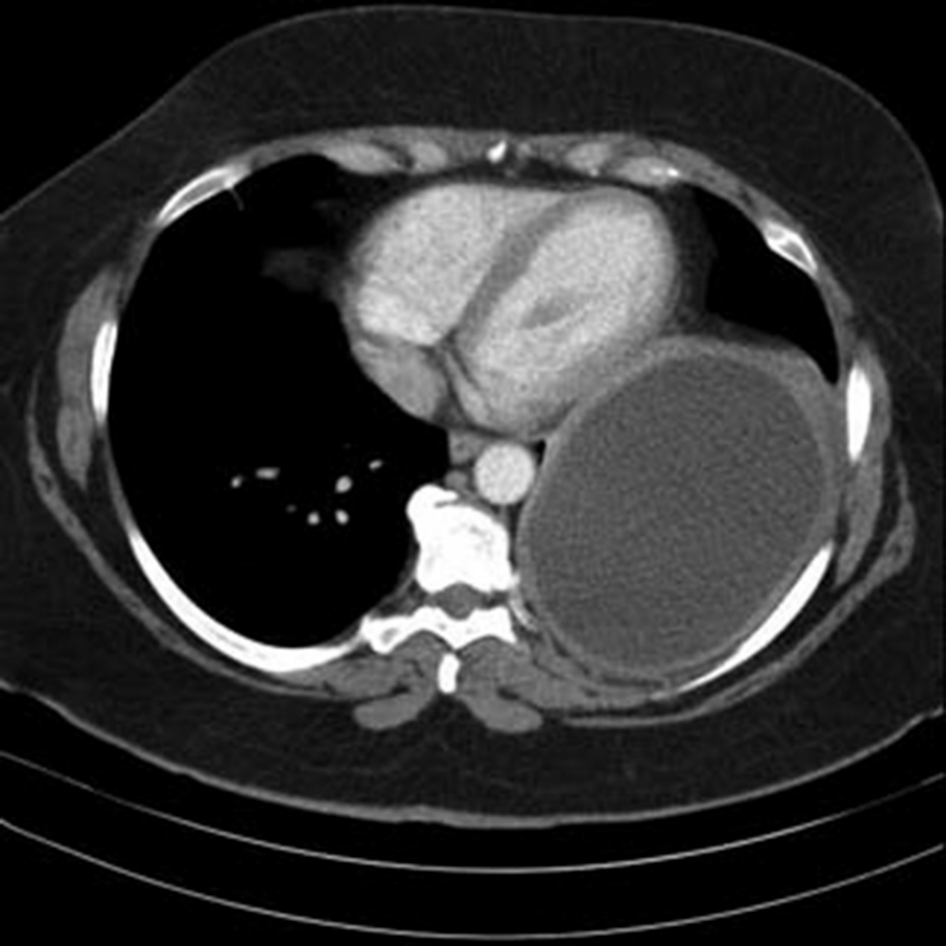
Chest CT-Scan

The patient was a 42 year old woman with pulmonary hydatid cyst who was admitted for left thoracotomy. She weighed about 110 kg and her body mass index (BMI) was about 42 kg/m^2^. She had a history of asthma since two years ago which was under treatment by inhaled Salbutamol and Seretide (PRN). In chest CT scan a large thick walled homogenous cystic lesion (12 cm, 5.8 cm, 8 cm) was seen in the left lower hemi-thorax (Figures 1 and 2).

**Figure 3.**
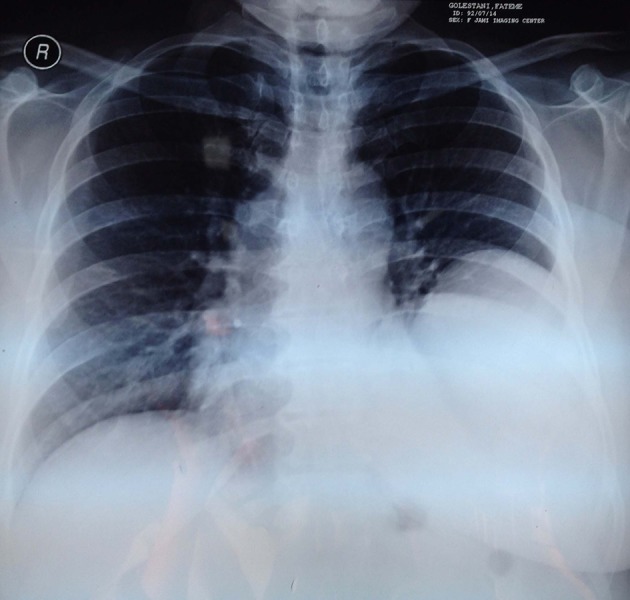
Chest Radiography on Admission

In trans-thoracic echocardiography, cardiac ejection fraction (EF) was reported about 55% with normal left and right ventricular size and function; however, a large cystic mass in postrolateral of pericardium was also noted. The chest radiography revealed a mass lesion with a homogenous shadow in the left lung (Figure 3).

Pulmonary function test (PFT) showed FEV1: 1.92 L, FEV1/FVC:82.6% and FVC: 2.32 L. All laboratory data (BUN, Cr, Na, K, CBC, FBS, AST, ALT) were normal. In the operating room and after placement of standard monitoring (ECG monitoring, pulse-oximetry, Non-invasive blood pressure monitoring) heart rate 80 beats/minute, blood pressure 136/75 mmHg and O_2_ saturation 98% in room air were observed. The right lung auscultation was clear and there was a reduction in left lung sounds. After premedication with Midazolam 2 mg/IV and Fentanyl 250 micg/IV general anesthesia was induced with Lidocaine 2% 100 mg/IV , Sodium thiopental 500 mg/IV and Cisatracurium 12 mg/IV and airway was secured with a Left-sided double lumen tube (No:35Fr). The correct position of endotracheal tube was confirmed by fiberoptic bronchoscopy and anesthesia was maintained with Isoflurane 1.2% MAC in 50% O_2_. After placement of Arterial catheter (from right radial artery) and central venous catheter (from right internal jugular), the patient was turned to right lateral decubitus position and the surgery was initiated. After about an hour of surgery, cyst aspiration led to rupture and suddenly a severe hypotension occurred (drop in Systolic blood pressure down to 50 mmHg) and airway pressure rose (Peak inspiratory pressure rose to 60 cm H_2_O with tidal volume of 300 ml and Respiratory rate of 16/min.) Immediately, we notified the surgeon to stop the surgery and volatile anesthetic was discontinued. After administration of 2 mL of Epinephrine 1/100/000 the patient was turned to supine position and free fluid resuscitation was performed. Because of high probability of anaphylactic shock and also presence of refractory hypotension, infusion of epinephrine 0.03-0.05 micg/kg/minutes started and hydrocortisone 300 mg/IV, Ranitidine 50 mg/IV and Salbutamol 4 puff via spacer through ETT was administered to the patient. Although that the left lung was isolated by double lumen tube, multiple and intermittent tracheal suctioning was done to lessen escolex exposure. Meanwhile, we recognized generalized skin rash on upper extremities and wheezing in lung auscultation. It took about an hour to stabilize the patient and then the surgery continued. At the end of the procedure, double lumen tube was change to single lumen endotracheal tube and the patient was transferred to intensive care unit (ICU). After about 12 hours of ICU observation, patient was successfully extubated. She was transferred to ward with stable hemodynamic two days later.

## 3. Discussion

The Hydatid cyst is a parasitic infection caused by the larva of *E. granulosis* (2-6). The adult tapeworm is about 3-6 mm long that lives in small intestine of definite hosts such as dogs and other canidae. Its eggs (if ingested) can cause infection in intermediate host like sheep, camel and goats. After the ingestion of fecal matter of definite host, human can be infected accidentally (2). Cyst can grow in different organs. In the lung, the most common site of involvement is the lower lobe of right lung and it is usually solitary (1). Calcification of pulmonary cysts are not usual and daughter cyst formation is rare (4). Rupture of pulmonary hydatid cyst in an awake individual, may cause chest pain (49%), dyspnea (42%), hemoptysis (33%) and sputum production (33%). However, under general anesthesia, the dominant signs are usually hypotension, tachycardia, arrhythmia, rash and urticaria (usually on the neck, face, upper extremities and anterior of the chest). Bronchospasm is less frequent especially after general anesthesia (3); although it was present in our case. During surgery, other substances such as muscle relaxants and antibiotics can cause anaphylaxis .We excluded them by the time of administration. In a study by Yimei *et al.* on demographic and clinical characteristics of patients with anaphylactic shock after surgery for cystic echinococcosis, most patients with hydatid cyst and anaphylactic shock were young and most of the lesions were in the lung. They postulated that different immune reactions to allergens (different amount of IgG and IgE production) are the possible reason (6). In our case, preoperative awareness of probability of anaphylactic shock, good and sufficient venous access and early resuscitation were the key components of successful management. As it has been emphasized in most guidelines, epinephrine is the vasopressor of choice during anaphylactic shock (3). Although the effects of glucocorticoids are delayed but we used them in acute setting to prevent the recurrence of manifestations in the late phase of anaphylaxis (3). From the preventive point of view, avoidance of the over-distention of the cyst by soft injection of scolicide and also its gentle manipulation can prevent anaphylactic reactions during surgery (3). According to a prospective study, preoperative administration of H1 and H2 receptor blockers were able to attenuate hemodynamic response of the rupture of hydatid cyst (3). However, it is still controversial and we used ranitidine after the occurrence of anaphylactic manifestations.

In conclusion, during the surgery of hydatid cyst, any hemodynamic instability should raise the suspension of anaphylaxis and early resuscitation should be instituted with the use of glucocorticoids, H1 and H2 receptor blockers, proper vasopressor and crystalloids.
